# Is health care a necessary or luxury product for Asian countries? An answer using panel approach

**DOI:** 10.1186/s13561-017-0144-8

**Published:** 2017-01-25

**Authors:** S. M. Abdullah, Salina Siddiqua, Rumana Huque

**Affiliations:** 1 Department of Economics, University of Dhaka, Dhaka, 1000 Bangladesh; 2 Department of Development Studies, University of Dhaka, Dhaka, 1000 Bangladesh

**Keywords:** Income elasticity, Health care expenditure, Panel cointegration, Panel unit root, Cross section dependence, FMOLS, DOLS, I10, C51

## Abstract

A number of studies have estimated the income elasticity of health care expenditure to identify whether health care is a necessary or luxury product. However, the issue has received less attention in developing countries, especially in Asian economies. The current study for the first time has used the panel data covering 36 Asian countries for the period 1995–2013 for revealing the nature of health care as a product. Along with conventional econometric techniques we have addressed the issue of cross section dependence and used Westerlund (2007) panel cointegration test which is robust against cross section dependence and heterogeneity for detecting the presence of panel cointegration. By applying Fully Modified OLS (FMOLS) and Dynamic OLS (DOLS) it was found that the long run elasticity of Health Care Expenditure (HCE) with Gross Domestic Product (GDP) is less than unit implying that the health care can be regarded as necessary in nature for these countries.

## Background

Macro level health spending has significant beneficial effects on health outcomes [1–3]. Data from 47 African countries [4] and 133 low and middle income countries [1] showed that increased health spending led to reduced infant and under-5 child mortality rates. Hence, the share of health expenditure, the determinants of resources a country devotes to medical care, and the relationship between national income and national health care expenditures have drawn attention of health economists worldwide. A large number of studies have estimated the income elasticity of health care expenditure to identify whether health care is a necessary or luxury product, and found varying results. While income elasticity coefficient of health care expenditure was found unity in 21 Organization of Economic Cooperation and Development (OECD) countries reflecting health care not a luxury [5], it was found less than unity in 13 MENA (Middle East and North Africa) countries characterizing health care as a necessary product [6]. However, majority of these studies have focused mainly on developed countries, including OECD countries [5, 7–9], United States [10], Middle East and African countries [6, 11], and European Countries [12, 13].^1^ The issue has been discussed little in developing countries, especially in Asian economies. In recent years only Hassan et. al [14] analysed this relation using South Asian Association for Regional Cooperation (SAARC) countries’ experience. One major limitation of many of the previous studies is that of using a single year of data to obtain cross-country estimates of the natural correlation [11, 15–17], making the regression results spurious. More recently, researchers have used panel data on Health Care Expenditure (HCE) and Gross Domestic Product (GDP) measured across countries and across time [18–23], which offers a number of advantages over cross-sectional study. Using multiple years of data enables researchers to include country-specific fixed effects for each country, thereby controlling for a wide range of time-invariant country characteristics, This avoids potential bias for the estimated relationship between HCE and GDP while retaining the cross sectional dependence and panel heterogeneity as important issues. In addition, the stationarity of the concerned variables are of vital importance when dealing with long panel. Considering these drawbacks of earlier studies, this study examined the impact of income on health care expenditure at macro level using a long panel of 36 Asian countries^2^ for the period 1995 to 2013 while addressing the issue of heterogeneity and cross sectional dependence.

The paper is organized in the following sections: section 2 describes the source of data and methodological procedures applied in this paper. In Section 3, results of econometric estimations have been discussed and section 4 concludes.

## Methods

We aimed to estimate income elasticity of health care expenditure and detect whether health care is a necessary or luxury product in Asian countries. Thus, we estimated the responsiveness of health care expenditure with respect to the change in income while controlling for other variables related to health status improvement. We estimated the following model by using appropriate econometric methods such as Fully Modified OLS (FMOLS) and Dynamic OLS (DOLS):

$$ lnHC{E}_{i,t}={\beta}_0+{\beta}_1lnGD{P}_{i,t}+{\beta}_2HS{I}_{i,t}+{\varepsilon}_{i,t} $$

where, i = 1, 2, −----- N and t = 1, 2, −----- T indexes cross section and time series units respectively. *lnHCE*_*i*,*t*_ is the natural logarithm of health care expenditure (measured in current US $) for country i at time t and *lnGDP*_*i*,*t*_ is the natural logarithm of GDP (measured in current US $) and used as proxy of income. *HSI*_*i*,*t*_ stands for health status improvement of country i at time t which is proxied by using infant mortality and life expectancy for all countries. Finally, *ε*_*i*,*t*_ is the error term with all unobserved factors.

In the above model, *β*_1_ is the coefficient which measures the impact of income on health care expenditure. As the higher the income the higher is the health care expenditure, this coefficient is expected to carry a positive sign. However, its magnitude determines whether health care is a necessary or luxury product. More specifically, as the standard theory of microeconomics suggests if,

$$ 0\le \frac{\delta lnHC{E}_{i,t}}{\delta lnGD{P}_{i,t}}={\beta}_1\le 1,thenhealthcarewouldbenecessaryproduct $$

$$ \frac{\delta lnHC{E}_{i,t}}{\delta lnGD{P}_{i,t}}={\beta}_1>1,thenhealthcarewouldbeluxuryproduct $$

### Data and statistical software

We have used two secondary data sources to develop the panel data set, namely World Development Indicators (WDI) and Health Nutrition and Population Statistics (HNPS) from World Bank database. A total of 36 Asian countries have been studied for the period of 1995 to 2013. The variables that we have used are, Health Care Expenditure, Gross Domestic Product and Health Status Improvement measured through Infant Mortality (IM) and Life Expectancy (LE). We used EViews 9 and STATA 14 softwares to carry out the econometric analysis.

### Cross section dependence test

The existence of common shocks among sample countries could result in creating contemporaneous correlation, popularly known as cross section dependency, among the different countries included in the panel. As the size of the panel unit root test usually becomes distorted because of the presence of cross sectional dependence, it is crucial to diagnose this issue before estimating panel data models. We tested the following null hypothesis: the residuals from the standard panel regression are contemporaneously uncorrelated. Therefore, we diagnosed whether the pair - wise covariance among residuals are zero or not. Symbolically:

$$ {H}_0:{\rho}_{ij}={\rho}_{ji}=Cov\left({\varepsilon}_{it},\ {\varepsilon}_{jt}\right)=0,\ for\  all\ t,\ i\ \ne j $$

$$ {H}_1:{\rho}_{ij}={\rho}_{ji}=Cov\left({\varepsilon}_{it},{\varepsilon}_{jt}\right)\ne 0,\ for\  all\ t,\ i\ \ne j $$

In order to test the above hypothesis, we applied four different tests namely Breuch – Pagan Lagrange Multiplier (LM) [24], Pesaran Cross Sectional Dependence (CD), Pesaran Scaled LM [25] and Baltagi, Feng and Kao Bias Corrected Sclaed LM [26]. However, Pesaran CD is regarded as the most general one as it is suitable for stationary and as well as non – stationary panels. It also consists of reasonable small sample properties.

### Panel unit root test

Panel based unit root test are more preferred to individual time series ones given the better power properties of the former test. Cross – sectional independence is a crucial assumption for all the readily available panel unit root tests. However, Im, Pesaran and Shin (IPS) panel unit root test by Im et al. [27] relaxes the restrictive assumptions of no serial correlation and panel homogeneity. Im et al. [28] proposed demeaning procedure (subtracting group mean from the data) to denounce the contemporaneous correlation of the data. Therefore, we have used IPS panel unit root test along with Levin, Lin and Chu (LLC) panel unit root test by Levin et al. [29], ADF – Fisher and PP – Fisher panel unit root test by Choi [30] to detect the stationarity of the variables. In order to carry out these tests first for each variable, we estimated an auroregressive (AR) process, and then an Augmented Dickey Fuller (ADF) test regression was fitted for each cross-section unit. For detecting the stationarity of the variables, the statistical significance of the autoregressive coefficient attached with lagged level dependent variable in test regression is tested. Therefore, if the ADF test regression takes the following form:

$$ \Delta {y}_{i,t}=\alpha {y}_{i,t-1}+{\displaystyle \sum_{j=1}^{p_i}}{\beta}_{i,j}\Delta {y}_{i,t-j}+{x}_{i,t}^{{\textstyle \hbox{'}}}\delta +{\varepsilon}_{i,t} $$

then the appropriate null hypothesis for testing the panel unit root would be *H*_0_ : *α*_*i*_ = 0, *for all i*. In the above regression *y*_*it*_ denotes the variable of concern for which stationary property would be tested and *x*_*i,t*_ stands for other control variables.

### Panel cointegration test

Several panel cointegration tests are available, including Pedroni [31, 32], McCoskey and Kao [33], Kao [34] and Westerlund [35]. We employed Pedroni [31, 32] and Westerlund [35] panel cointegration test for detecting the existence of cointegrating relationship among the variables. The reason is that the first one allows for heterogeneity while the second one is robust against heterogeneity and cross correlation in panel.

### Pedroni panel coinetgration test

The Pedroni [31, 32] is an Engle – Granger based panel cointegration test. Pedroni’s proposed test for panel cointegration considers heterogeneous intercept and trend coefficient across cross country. We estimated the following regression to conduct the test:

$$ HC{E}_{i,t}={\alpha}_i+{\phi}_it+{\gamma}_1GD{P}_{i,t}+{\gamma}_2HS{I}_{i,t}+{e}_{i,t} $$

where, i = 1, 2, −----- N and t = 1, 2, −----- T, and HCE, GDP and variables i.e. infant mortality and life expectancy under HSI are assumed to be integrated of order one, I(1). We obtained the residuals from the above regression and performed an ADF test on the residuals to estimate whether they are I(1) using the following test regression for each country:

$$ \varDelta {e}_{i,t}={\rho}_{it}{e}_{i,t-1}+{\displaystyle \sum_{j=1}^{p_i}}{\psi}_{i,j}\varDelta {e}_{i,t-1}+{v}_{i,t} $$

Based on various methods, Pedroni provided a total of eleven statistics in two groups; panel statistic (within dimension) and group statistic (between dimension). The following hypothesis is tested against the alternatives:

$$ {H}_0:\ {\rho}_i=0\ \left( No\  Cointegration\right) $$

$$ \begin{array}{l} Homogeneous\  Alternative,\ {H}_1:\left({\rho}_i = \rho \right)<1\ \forall\ i\ \\ {} Heterogeneous\kern0.5em  Alternative,\ {H}_1:{\rho}_i<1\ \forall\ i\end{array} $$

In particular panel statistic is concerned with homogeneous alternative while group statistics is concerned with the other. However, all these statistics are distributed as asymptotically normal.

### Westerlund panel cointegration test

There are cases where theory suggests existence of long run relationship among variables, panel cointegration tests have failed to reject the null hypothesis of *“no cointegration”*. It occurs due to the failure of *“common – factor restriction”* (Banerjee et. al [36]). By depending on structural dynamics (rather than error dynamics) Westrelund [35] have developed a set of panel cointegration test which do not require any common factor restriction. These tests are general enough to be robust against heterogeneity and cross section dependence. The cointegration test assumes the following data generating process:

$$ \varDelta HC{E}_{i,t}={\delta}_i^{{\textstyle \hbox{'}}}{d}_t+{\alpha}_iHC{E}_{i,t-1}+{\lambda}_i^{{\textstyle \hbox{'}}}{x}_{i,t-1}+{\displaystyle \sum_{j=1}^{p_i}}{\alpha}_{ij}\varDelta HC{E}_{i,t-1}+{\displaystyle \sum_{j=-{q}_i}^{p_i}}{\gamma}_{ij}\varDelta {x}_{i,t-1}+{e}_{i,t} $$

Where, i = 1, 2, −----- N and t = 1, 2, −----- T indexes cross sectional units and time series. In the above process, *x* is a vector of independent variables that includes GDP and HSI measured in terms of infant mortality and life expectancy and finally *d*_*t*_ contains deterministic components. Here, *α*_*i*_ is referred to as error correction parameter. If *α*_*i*_ < 0 then there will be error correction and hence cointegration while if *α*_*i*_ = 0, reflects that the error correction is absent and consequently there is no cointegration.

Westerlund [35] proposed four panel statistics, among which two (Panel Statistic) test the alternative hypothesis that panel is cointegrated as a whole while the other two (Group Statistic) test that at least one is cointegrated. Thus, the null and alternative hypothesis tested is formulated in the following way:

$$ Panel\  Statistic:\ {H}_0:{\alpha}_i=0\  against\ {H}_1^P:\ {\alpha}_i<0\ \forall\ i $$

$$ Group\  Statistic:\ {H}_0:{\alpha}_i=0\  against\ {H}_1^G:\ {\alpha}_i<0\ for\  at\  least\  some\ i $$

### Estimation of cointegrating relationship

In our data GDP and HSI i.e. infant mortality and life expectancy can become endogenous and also the error terms can be serially correlated which would result in the dependence of OLS estimators on nuisance parameters. In order to solve these problems two estimators namely FMOLS and DOLS can be introduced. Phillips and Hansen [37] proposed a semi parametric correction for the problem of long run correlation among cointegrating equation and stochastic regressors innovations resulting in FMOLS estimators. It is asymptotically unbiased. On the other hand Saikkonen [38] and Stock and Watson [39] advanced an asymptotically efficient estimator which eliminates the feedback in the cointegrating system by augmenting the cointegrating regression with lags and leads of independent variables. The resulting estimator is known as DOLS estimator.

With a view to explain the idea of FMOLS estimator consider the following fixed effect model:

$$ HC{E}_{i,t}={\alpha}_i+{x}_{i,t}^{\hbox{'}}\beta +{u}_{i,t} $$

Where, i = 1, 2,-----, N and t = 1, 2, −----, T indexes cross section and time series units respectively, *HCE*_*i*,*t*_ is the health care expenditure (an I(1) process), *β* is (2*1) vector of parameters, *α*_*i*_ are intercepts and *u*_*i*,*t*_ are the stationary disturbance terms. Here *x*_*i*,*t*_ are assumed to be (2*1) vector of independent variables (GDP and HSI measured with infant mortality and life expectancy) which are I(1) for all cross section units. It is assumed that it follows an autoregressive process of following form:

$$ {x}_{i,t}={x}_{i,t-1}+{\varepsilon}_{i,t} $$

$$ Innovation\  Vector,\ {w}_{i,t}=\left({u}_{i,t},\ {\varepsilon}_{i,t}\right) $$

Given that *w*_*i*,*t*_ = (*u*_*i*,*t*_, *ε*_*i*,*t*_) ~ *I*(0) the variables are said to be cointegrated for each members of the panel with cointegrating vector *β*. The asymptotic distribution of the OLS estimator is condition to the long run covariance matrix of the innovation vector. The FMOLS estimator is derived by making endogeneity correction (by modifying variable *HCE*_*i*,*t*_) and serial correlation correction (by modifying long run covariance of innovation vector, *w*_*i*,*t*_). The resulting final estimator is expressed as follows:

$$ {\widehat{\beta}}_{FMOLS} = {\left[{\displaystyle \sum_{i=1}^N}{\displaystyle \sum_{t=1}^T}\left({x}_{it}-{\overline{x}}_i\right){\left({x}_{it}-{\overline{x}}_i\right)}^{\hbox{'}}\right]}^{-1}*\left[{\displaystyle \sum_{i=1}^N}\left({\displaystyle \sum_{t=1}^T}\left({x}_{it}-{\overline{x}}_i\right){\widehat{HCE}}_{it}-T{\widehat{\varDelta}}_{\varepsilon u}\right)\right] $$

The cointegrating regression is augmented by lead and lagged differences of GDP and other independent variables in DOLS framework for controlling endogeneity (by following Saikkonen, [38]). For controlling serial correlation, lead and lagged difference of the HCE has also been included in the model (by following Stock and Watson, [39]). Thus, the estimated regression equation under DOLS framework was as follows:

$$ HC{E}_{i,t}={\alpha}_i+{\beta}_i{x}_{i,t}+{\displaystyle \sum_{k=-{p}_1}^{p_2}}{\delta}_k\varDelta HC{E}_{i,t-k}+{\displaystyle \sum_{k=-{q}_1}^{q_2}}{\lambda}_k\varDelta {x}_{i,t-k}+{u}_{i,t} $$

## Results

With a view to determining the appropriate estimation method, we need to check the stationary of the variables and also their order of integration. However, cross sectional dependence or cross sectional correlation of the variables is a fact that should be detected for the variables to decide which panel unit root test would be applied.

Table 1 contains the test results for Cross Sectional Dependence of different variables, and suggests that it is possible to reject the null hypothesis for all the variables at 1% level of significance. Therefore, the residuals from the standard panel regression would be contemporaneously correlated and this should be addressed while panel stationarity would be tested.

**Table 1:** Test Results for Cross Sectional Dependence of the Variables

Variables and Test Names		Breusch - Pagan LM	Pesaran - Scaled LM	Bias Corrected Scaled LM	Pesarn CD
*H*_*0*_ *: No Cross - Section Dependence*					
GDP (Current US $)	Statistic	10994.69^a^	291.99^a^	290.99^a^	104.58^a^
	Prob.	0.000	0.000	0.000	0.000
Health Care Expenditure (Current US $)	Statistic	10541.35^a^	279.22^a^	278.22^a^	102.40^a^
	Prob.	0.000	0.000	0.000	0.000
Infant Mortality	Statistic	10563.79^a^	279.85^a^	278.85^a^	100.50^a^
	Prob.	0.000	0.000	0.000	0.000
Life Expectancy	Statistic	11418.19^a^	303.92^a^	302.92^a^	106.79^a^
	Prob.	0.000	0.000	0.000	0.000

Note: ^a^ Indicates 1% level of significance

Therefore, we have used IPS panel unit root test to detect the stationarity of the variables along with some other tests e.g. Levin, Lin and Chu (LLC) test (Levin et al., [29]), ADF - Fisher Test and PP – Fisher test (Choi, [30]).

Table 2 and Tables 7, 8 and 9 in Appendix contain the panel unit root test results for each of the variables. All the tests are concerned with the null hypothesis of *“Panels Contain Individual Unit Root”* except LLC that tests the null hypothesis of *“Panel Contains Common Unit Root”*. The tests have been carried out with two different test regression specifications; one with constant and the other with constant and trend. It is evident from the test results that GDP and HCE are difference stationary i.e. I(1) variable according to all tests. With regards to infant mortality, it is also found to be difference stationary in intercept and trend specification under IPS, ADF – Fisher and PP – Fisher test. Thus it can be treated as an I(1) variable. Life expectancy was found to be difference stationary in intercept specification under IPS, in intercept and trend specification under LLC and in both specification under ADF - Fisher test. Since all the variables have been found to be integrated of a unique order we have identified the long run relationship among them by establishing the panel cointegration.

**Table 2:** Panel Unit Root Test Results of the Variables

Variables	Im – Pesaran – Shin (IPS) Test for Panel Unit Root			
	*Null: Panels Contain Unit Roots(Individual)*			
	Intercept		Intercept and Trend	
	IPS W – Stat	Prob.	IPS W - Stat	Prob.
GDP (Current US $)	20.06	1.000	6.15	1.000
D(GDP)	−4.79^a^	0.000	−12.43^a^	0.000
Health Care Expenditure (Current US $)	21.23	1.000	6.07	1.000
D(Health Care Expenditure)	−3.70^a^	0.000	−12.61^a^	0.000
Infant Mortality	−20.81^a^	0.000	0.36	0.642
D(Infant Mortality)	−2.18	0.014	−5.04^a^	0.000
Life Expectancy	−1.62	0.052	−10.72^a^	0.000
D(Life Expectancy)	−15.08^a^	0.000	−20.25^a^	0.000

Note: ^a^Indicates 1% level of significance

In order to check the existence of cointegration among the variables along with Pedroni [31, 32] Engle Granger based panel cointegration test, we applied Westerlund [35] error correction based panel cointegration test. The later one is already established in the literature for its robustness against panel with heterogeneity and cross sectional dependence. Hence, application of this test allowed us to check issue of existence of cointegartion among health care expenditure and income while controlling for health status improvement measured with infant mortality and life expectancy in a more comprehensive manner. Both the tests have been performed with three different deterministic specifications.

Table 3 and Table 10 in Appendix contain the test results of panel cointegration. For testing the cointegration among health care expenditure, income and infant mortaility when neither constant nor trend was used as deterministic specification, the null hypothesis of *“no cointegration”* was rejected by all 11 statistic under Pedroni [31, 32] test. All the 4 statistic of Westerlund [35] test have also found to be significant. In the same specification when cointegration was checked among health care expenditure, income and life expectancy a total of 7 statistic in Pedroni [31, 32] and 2 among 4 statistic of Westerlund [35] was found to be statistically significant. When the deterministic specification was changed to allow the presence of constant, only 7 and 6 of 11 statistic for infant mortality and life expectancy respectively have found to be able to reject the non existence of cointegration under Pedroni [31, 32] test. By using the similar deterministic specification Westerlund [35] test was able to reject the null for both variables under 2 statistic out of 4. The conclusion of Westerlund [35] test has remained the same when the deterministic specification allows for both the constant and trend. While Pedroni [31, 32] test was carried out by using the later deterministic specification, 4 statistic have found to be significant when panel cointegration was checked with infant mortality while the number was 6 if it was tested with life expectancy. Thus, it can be argued that there might exists a long run cointegrating relationship among health expenditure, income and health status improvement which is substituted by using infant mortality and life expectancy.

**Table 3:** Westerlund Panel Cointegration Test

Westerlund (2007) ECM Panel Cointegration Test, *H*_*0*_ *: No Cointegration*				
	HCE, GDP and IM		HCE, GDP and LE	
Statistic	Stat.	Prob.	Stat.	Prob.
Deterministic Specification: No Constant & Trend				
Gt	−2.702^a^	0.000	−2.788^a^	0.000
Ga	−7.807^b^	0.015	−6.778	0.148
Pt	−16.120^a^	0.000	−12.352^a^	0.000
Pa	−11.134^a^	0.000	−2.579	0.461
Deterministic Specification: Constant Only				
Gt	−2.912^a^	0.000	−3.074^a^	0.000
Ga	−7.555	0.934	−6.475	0.994
Pt	−18.933^a^	0.000	−18.24^a^	0.000
Pa	−6.287	0.326	−6.512	0.244
Deterministic Specification: Constant & Trend				
Gt	−2.921^a^	0.003	−3.096^a^	0.000
Ga	−5.376	1.000	−4.293	1.000
Pt	−18.059^a^	0.000	−16.477^a^	0.001
Pa	−5.223	1.000	−5.665	1.000

Note: ^a^and ^b^Indicates 1% and 5% level of significance respectively

With a view to estimate the cointegrating vector we have applied two different methods, FMOLS and DOLS. Each of the methods provides three different estimators namely, pooled, pooled weighted and grouped mean. The pooled FMOLS estimator is the extension of Phillips and Hansen [37] FMOLS estimator offered by Phillips and Moon [40] which provides the estimators after correcting deterministic components in regressand and regressors. In order to allow different long run variances across the cross section for heterogeneous panels, Pedroni [41] and Kao and Chiang [42] proposed pooled weighted FMOLS. Finally the grouped mean FMOLS estimator developed by Pedroni [41, 43] is derived by averaging the individual cross section FMOLS estimates. In contrast to FMOLS, augmentation of model with lags and leads of differenced regressand and regressors in DOLS helps it to overcome the problem of asymptotic endogeneity and serial correlation. Kao and Chiang [42], Mark and Sul [44, 45] and Pedroni [43] extended the standard DOLS estimation developed by Saikkonen [38] and Stock and Watson [39]. Kao and Chiang [42] proposed pooled DOLS where the augmented cointegrating regression allows the short run dynamics to be cross section specific. By allowing heterogenous long run variance, Mark and Sul [44, 45] developed pooled weighted DOLS. Finally, Pedroni [43] developed the grouped mean DOLS estimates by averaging the individual cross section DOLS estimates.

Table 4 contains the estimation results of long run relationship between health care expenditure and GDP and infant mortality as a measure of health status improvement. It is evident that the elasticity of health care expenditure with respect to income in Asian countries is less than unit. The elasticity coefficient varies from 0.73 to 0.83 when the model was estimated using FMOLS while the range was found to be 0.67 to 0.80 while estimating using DOLS method. The Wald test was observed to be significant in all cases. Thus, health care in Asian countries can be argued as a necessary and normal product. The elasticity coefficient of infant mortality was found to be negative and significant in almost all cases. Therefore, there is an inverse relationship between health care expenditure and infant mortality in Asian countries. Thus, the higher the health care expenditure, the lower the infant mortality or vice – versa.

**Table 4:** Estimation of Cointegrating Regression with Infant Mortality as proxy for Health Status Improvement

Variables	FMOLS			DOLS		
	Pooled	Weighted	Grouped	Pooled	Weighted	Grouped
Cointegrating Regression						
Log of GDP	0.832^a^	0.730^a^	0.816^a^	0.742^a^	0.807^a^	0.676^a^
	(0.040)	(0.009)	(0.042)	(0.081)	(0.025)	(0.127)
Log of IM	−0.366^a^	−0.431^a^	−0.675^a^	−0.477^a^	−0.389^a^	−0.861
	(0.087)	(0.001)	(0.190)	(0.136)	(0.054)	(0.758)
Wald Test, H_0_: Coefficient of Log of GDP equal to One						
t – Stat.	−4.166^a^	−28.637^a^	−4.355^a^	−3.155^a^	−7.666^a^	−2.534^b^
*χ* ^2^ Stat.	17.357^a^	820.080^a^	18.973^a^	9.957^a^	58.782^a^	5.719^b^

Note: In DOLS (Pooled) and FMOLS (Pooled) estimation coefficient of covariance was computed using sandwich method and in DOLS (Grouped) estimation individual heteroscedasticity and autocorrelation consistent standard errors and covariances were used. Numbers in the parenthesis indicate standard error. ^a^ Indicates 1% and ^b^ indicates 5% level of significance

Table 5 contains the results of cointegrating relationship between health care expenditure and GDP and another indicator of health status improvement measured by life expectancy. The elasticity coefficient of health care expenditure with respect of income was also found to be less than one. Here the elasticity measure varied in between 0.84 to 0.92 in FMOLS method and 0.77 to 0.91 in DOLS method. The coefficient has been observed to remain positive and significant in all cases. Thus, for Asian countries health care expenditure increases less than proportionately with the increase in income. The coefficient of life expectancy was found to be significant in almost all cases. Therefore, with respect to increase in life expectancy, health care expenditure increases in these countries.

**Table 5:** Estimation of Cointegrating Regression with Life Expectancy as proxy for Health Status Improvement

Variables	FMOLS			DOLS		
	Pooled	Weighted	Grouped	Pooled	Weighted	Grouped
Cointegrating Regression						
Log of GDP	0.924^a^	0.841^a^	0.924^a^	0.774^a^	0.917^a^	0.831^a^
	(0.033)	(0.009)	(0.031)	(0.085)	(0.027)	(0.100)
Log of LE	0.912	1.489^a^	5.464^a^	3.906^a^	1.416^b^	10.419^a^
	(0.629)	(0.001)	(1.459)	(1.496)	(0.673)	(3.530)
Wald Test, H_0_: Coefficient of Log of GDP equal to One						
t – Stat.	−2.20^b^	−16.091^a^	−2.436^b^	−2.632^a^	−3.023^a^	−1.674^c^
*χ* ^2^ Stat.	4.883^b^	258.944^a^	5.938^b^	6.930^a^	9.140^a^	2.805^c^

Note: In DOLS (Pooled) and FMOLS (Pooled) estimation coefficient of covariance was computed using sandwich method and in DOLS (Grouped) estimation individual heteroscedasticity and autocorrelation consistent standard errors and covariances were used. Numbers in the parenthesis indicate standard error. ^a^ Indicates 1%, ^b^ indicates 5% and ^c^ indicates 10% level of significance

Thus, unlike many OECD and developed countries such as USA, Canada, Germany and Italy where health care expenditure has been identified as luxury good, for Asian Countries it is revealed to be a necessary one. The findings contradict Hassan et. al [14] but in line with what have been found in Dreger and Reimers [5], Mehrara et. al [6] and Penas et. al [8].

## Discussion and conclusion

By exploiting data for the period of 1995 to 2013, the study finds that long run elasticity of health care expenditure in relation to income is less than unit in 36 Asian countries, ensuring that the health care can be treated as a necessary product as a whole for the sample countries. In general the responsiveness was found to be higher when life expectancy was used instead of infant mortality as proxy of health status improvement. Our finding is different from that of Hassan et. al [14] which suggested health care as luxury products for South Asian Association for Regional Cooperation (SAARC) countries. However, the latter study has ignored the issue of cross correlation which may mislead the findings. Moreover, the way the coefficients had been analyzed made the reasoning and policy implications rather weak.

The contribution of this paper to the literature on the relationship between health care expenditure and income is twofold. First, it has covered almost all the countries in Asia, and analyzed the issue in a more rigorous manner in the sense of addressing cross correlation and heterogeneity problem that potentially exists in the panel and brought the findings in front to realize how health care should be treated in those countries. Second, from methodological point of view, as the study has addressed the issue of cross sectional correlation and panel heterogeneity, the findings are more reliable. The current work has examined the existence of long run relationship between health care expenditure and income using a panel cointegration technique which is robust against cross sectional correlation and panel heterogeneity along with conventional panel cointegration test. The estimation technique- FMOLS and as well as DOLS- has been used which is robust against asymptotic endogeneity and serial correlation.

The study has a number of areas to improve. As cross section dependence was detected it would have been better if second generation panel unit root tests were used when identifying the integration order of the variables. However, demeaning of data has taken care of the severity of problem to some extent. Throughout the analysis the parameters have been assumed to be stable, thus should there be any structural instability the findings may vary. Further research is required addressing the above issues.

## Appendix

**Table 6:** Summary of Existing Studies

Study	Data	Method	Elasticity of Income/Findings
Abel – Smith (1967) [15]	30 Countries from Africa, Europe, America, South East Asia, Eastern Mediterranean, Western Pacific, 1961	Cross – Sectional (Survey)	>1
Kleiman (1974) [51]	16 Countries, 1968		>1
Maxwell (1981) [52]	10 Countries, 1975		>1
Gertler & van der Gaag (1990) [48]	25 Countries, 1975		>1
Getzen (1990) [49]	United States, 1966–1987		>1
Schieber (1990) [53]	Canada, France, The Federal Republic of Germany, Italy, Japan, The United Kingdom, and The United States, 1960–1987	Country Specific Time Series, Compound Annual Rate of Growth	>1
Getzen & Poullier (1992) [50]	19 OECD Countries, 1965–1986	Country Specific Time Series, Forecasting Model and Coparison of Mean Absolute Error (MAE)	>1
Fogel (1999) [47]	United States	Long Term Trend Analysis	>1
Newhouse (1977) [66]	13 OECD Countries, 1968–1972	Cross – Sectional, Regression Model Estimation	>1
Leu (1986) [60]	19 OECD Countries	Cross – Sectional	>1
Parkin *et. al* (1987) [17]	18 OECD Countries, 1980	Cross – Sectional, Estimation of Linear, Semi – Log, Exponential and Double Log Model	>1
Gbesemete & Gerdtham (1992) [11]	30 African Countries, 1984	Cross – Sectional, WLS, RESET Test	Income elasticity close to unity
Hitiris & Posnett (1992) [21]	20 OECD Countries, 1960–1987	Panel Data, Estimation of Linear and Log Linear Models	Income elasticity close to unity
Gerdtham *et. al* (1992) [20]	19 OECD Countries, 1987	Cross Section, General to Specific Approach	>1
Hansen & King (1996) [55]	20 OECD Countries, 1960–1987	Time Series, Unit Root and Cointegration	HCE, GDP are Stationary
Blomqvist & Carter (1997) [58]	24 OECD Countries, 1960–1991	Time Series, OLS, Unit Root, Cointegration and Long Run Estimation	Reservation about having an elasticity coefficient as greater than 1
Hitiris (1997) [12]	10 European Community Countries (1960–1991)	Panel Data, GLS, Pooled OLS	>1
McCoskey & Selden (1998) [33]	20 OECD Countries	Panel Data, Panel unit Root	HCE, GDP are Stationary
Roberts (2000) [13]	10 European Community Countries (1960–1993)	Panel Data, GLS, Unit Root and Long Run Relationship	Short Run Income Elasticity is significantly less than Unity
Okunade & Murthy (2002) [56]	USA, 1960–1997	Time Series, Unit Root and Cointegration	>1
Bhat & Jain (2004) [59]	OECD Countries	Panel Data	>1
Clemente *et. al* (2004) [57]	22 OECD Countries	Country Specific Time Series, Cointegration and Long Run Analysis	>1
Sen (2005) [23]	15 OECD Countries, 1990–1998	Panel Data Fixed Effect Model, GLS, WLS and IV Estimation	Elasticity varied between 0.21 to 0.51
Dreger & Reimers (2005) [5]	21 OECD Countries, 1975–2001	Panel Unit Root and Panel Cointegration	Health Care Expenditures are not Luxury Good. Income elasticity is not different from unity.
Wang & Rettenmaier (2006) [61]	50 States in the United States, 1980–2000	Panel Data, Unit Root with Structural Break, Cointegration	>1
Chakroun (2009) [54]	17 OECD Countries, 1975–2003	Panel Threshold Regression	Health Care is a Necessity rather than a Luxury
Baltagi & Moscone (2010) [18]	20 OECD Countries 1971–2004	Panel Data, Panel Unit Root, Corss Section Dependence, Common Correlated Effect Pooled Estimator (CCEP), Panel Fixed Effect, Spatial MLE	<1
Mehrara *et. al* (2012) [6]	13 MENA (Middle East & North Africa), 1995–2005	Panel Data, Panel Unit Root and Panel Cointegration	<1
Liu et. al (2011) [22]	22 OECD Countries, 1960–2002	Semiparametric Panel Varying Coefficient Model	Relationship between HCE and Income is subject to Structural Change. Full Sample Income Elasticity was greater than unity.
Hassan *et. al* (2014) [14]	South Asian Association for Regional Cooperation (SAARC), 1995–2010	Panel Data, Panel Unit Root Test, Panel Cointegration, Panel Long Run Estimation	HCE are Luxury Goods in SAARC Countries
Penas *et. al* (2013)	31 OECD Countries, 1970–2009	Panel Data, Panel Unit Root, LSDV and NLLS Estimation	Difference in Short term and Long Term Elasticities, Long run Elasticity is close to unity
Jewell *et. al* (2003) [7]	20 OECD Countries, 1960–1997	Panel Data, Panel Unit Root Test with Structural Breaks	HCE, GDP are Stationary when allowing for Structural Breaks

Note: Adapted and Extended by using, Getzen [46], Penas *et. al* [8] and Mehrara *et. al* [6]

**Table 7:** Panel Unit Root Test Results of the Variables

Variables	Levin, Lin & Chu Test for Panel Unit Root			
	*Null: Panels Contain Unit Roots(Common)*			
	Intercept		Intercept and Trend	
	LLC t – Stat	Prob.	LLC t - Stat	Prob.
GDP (Current US $)	17.35	1.000	−1.23	0.1090
D(GDP)	−6.36^a^	0.000	−14.12^a^	0.000
Health Care Expenditure (Current US $)	20.28	1.000	−0.43	0.332
D(Health Care Expenditure)	−4.98^a^	0.000	−10.69^a^	0.000
Infant Mortality	−32.60^a^	0.000	−3.92^a^	0.000
D(Infant Mortality)	−3.49^a^	0.000	−7.52^a^	0.000
Life Expectancy	−4.98^a^	0.000	−1.54	0.060
D(Life Expectancy)	−12.32^a^	0.000	−19.43^a^	0.000

Note: ^a^Indicates 1% level of significance

**Table 8:** Panel Unit Root Test Results of the Variables

Variables	ADF Fisher Test for Panel Unit Root (Choi, 2001)			
	*Null: Panels Contain Unit Roots(Individual)*			
	Intercept		Intercept and Trend	
	ADF – Chi Z - Stat	Prob.	ADF – Choi Z - Stat	Prob.
GDP (Current US $)	17.79	1.000	6.66	1.000
D(GDP)	−4.45^a^	0.000	−11.03^a^	0.000
Health Care Expenditure (Current US $)	18.40	1.000	7.02	1.000
D(Health Care Expenditure)	−3.15^a^	0.000	−11.13^a^	0.000
Infant Mortality	−14.46^a^	0.000	2.39	0.991
D(Infant Mortality)	−1.46	0.072	−3.78^a^	0.000
Life Expectancy	−2.18	0.014	−1.15	0.123
D(Life Expectancy)	−11.65^a^	0.000	−13.95^a^	0.000

Note: ^a^Indicates 1% level of significance

**Table 9:** Panel Unit Root Test Results of the Variables

Variables	PP- Fisher Test for Panel Unit Root (Choi, 2001)			
	*Null: Panels Contain Unit Roots(Individual)*			
	Intercept		Intercept and Trend	
	PP – Chi Z - Stat	Prob.	PP – Choi Z - Stat	Prob.
GDP (Current US $)	21.15	1.000	8.75	1.000
D(GDP)	−7.78^a^	0.000	−14.65^a^	0.000
Health Care Expenditure (Current US $)	21.33	1.000	10.34	1.000
D(Health Care Expenditure)	−7.59^a^	0.000	−13.89^a^	0.000
Infant Mortality	−23.52^a^	0.000	3.90	1.000
D(Infant Mortality)	−2.46^a^	0.006	−4.94^a^	0.000
Life Expectancy	−7.97^a^	0.000	4.74	1.000
D(Life Expectancy)	−1.926	0.027	1.84	0.967

Note: ^a^Indicates 1% level of significance

**Table 10:** Pedroni Panel Cointegration Test

Pedroni (1999, 2004) Engle – Granger Based Cointegration Test, *H*_*0*_ *: No Cointegration*						
Panel Statistic					Group Statistic	
	IM		LE		IM	LE
	Stat.	W-Stat	Stat.	W-Stat	Stat.	Stat.
Deterministic Specification: No Constant & Trend						
V-Stat	3.069^a^	1.629^a^	−2.61	0.407	-	-
	(0.001)	(0.051)	(0.995)	(0.341)	-	-
Rho-Stat	−4.214^a^	−3.116^a^	−0.372	−2.349^a^	−1.659^b^	−1.227
	(0.000)	(0.000)	(0.354)	(0.009)	(0.048)	(0.109)
PP-Stat	−5.164^a^	−5.469^a^	−4.595^a^	−4.942^a^	−7.727^a^	−8.896^a^
	(0.000)	(0.000)	(0.000)	(0.000)	(0.000)	(0.000)
ADF-Stat	−4.58^a^	−6.337^a^	−4.582^a^	−5.999^a^	−8.271^a^	−9.302^a^
	(0.000)	(0.000)	(0.000)	(0.000)	(0.000)	(0.000)
Deterministic Specification: Constant Only						
V-Stat	0.573	−0.024	2.113^b^	−0.061	-	-
	(0.283)	(0.509)	(0.017)	(0.524)	-	-
Rho-Stat	−0.701	−1.813^b^	0.071	−1.57^c^	0.093	0.292
	(0.241)	(0.034)	(0.528)	(0.058)	(0.537)	(0.615)
PP-Stat	−1.826^b^	−6.580^a^	−1.015	−6.389^a^	−7.881^a^	−8.119^a^
	(0.033)	(0.000)	(0.154)	(0.000)	(0.000)	(0.000)
ADF-Stat	−1.328^c^	−9.675^a^	−1.129	−8.969^a^	−11.31^a^	−10.507^a^
	(0.092)	(0.000)	(0.129)	(0.000)	(0.000)	(0.000)
Deterministic Specification: Constant & Trend						
V-Stat	−2.933	−3.527	2.113^b^	−0.061	-	-
	(0.998)	(0.999)	(0.017)	(0.524)	-	-
Rho-Stat	3.022	1.37	0.071	−1.57^b^	3.023	0.292
	(0.998)	(0.914)	(0.528)	(0.058)	(0.998)	(0.615)
PP-Stat	0.316	−5.997^a^	−1.015	−6.389^a^	−8.391^a^	−8.119^a^
	(0.624)	(0.000)	(0.154)	(0.000)	(0.000)	(0.000)
ADF-Stat	0.653	−8.513^a^	−1.129	−8.969^a^	−8.197^a^	−10.507^a^
	(0.743)	(0.000)	(0.124)	(0.000)	(0.000)	(0.000)

Note: ^a^,^b^ and ^c^ Indicates 1%, 5% and 10% level of significance respectively

## Footnotes

1 A summary of previous studies have presented in Table 6 in Appendix

2 List of Countries: Bahrain, Bangladesh, Bhutan, Brunei, Cambodia, China, India, Indonesia, Iran, Israel, Japan, Jordan, Kazakhstan, Korea Rep., Kuwait, Kyrgyzstan, Laos, Lebanon, Malaysia, Mongolia, Maldives, Nepal, Oman, Pakistan, Philippines, Qatar, Saudi Arabia, Singapore, Srilanka, Tajikistan, Thailand, Turkmenistan, United Arab Emirates, Uzbekistan, Vietnam and Yemen
